# Country-wide assessment of the genetic polymorphism in *Plasmodium falciparum *and *Plasmodium vivax *antigens detected with rapid diagnostic tests for malaria

**DOI:** 10.1186/1475-2875-7-219

**Published:** 2008-10-28

**Authors:** Natacha Mariette, Céline Barnadas, Christiane Bouchier, Magali Tichit, Didier Ménard

**Affiliations:** 1 Malaria Research Unit, Institut Pasteur de Madagascar, Antananarivo, Madagascar; 2 Plate-forme Génomique, Institut Pasteur de Paris, Paris, France

## Abstract

**Background:**

Rapid diagnostic tests (RDTs) are becoming increasingly indispensable in malaria management, as a means of increasing the accuracy of diagnosis. The WHO has issued recommendations, but the selection of the most suitable RDT remains difficult for users in endemic countries. The genetic variability of the antigens detected with RDTs has been little studied, but may affect the sensitivity of RDTs. This factor has been studied by comparisons between countries at continental level, but little information is available concerning antigen variability within a given country.

**Methods:**

A country-wide assessment of polymorphism of the PfHRP2, PfHRP3, pLDH and aldolase antigens was carried out in 260 *Plasmodium falciparum *and 127 *Plasmodium vivax *isolates, by sequencing the genes encoding these antigens in parasites originating from the various epidemiological strata for malaria in Madagascar.

**Results:**

Higher levels of polymorphism were observed for the *pfhrp2 *and *pfhrp3 *genes than for the *P. falciparum *and *P. vivax aldolase *and *pldh *genes. *Pfhrp2 *sequence analysis predicted that 9% of Malagasy isolates would not be detected at parasite densities ≤ 250 parasites/μl (ranging from 6% in the north to 14% in the south), although RDTs based on PfHRP2 detection are now recommended in Madagascar.

**Conclusion:**

These findings highlight the importance of training of health workers and the end users of RDTs in the provision of information about the possibility of false-negative results for patients with clinical symptoms of malaria, particularly in the south of Madagascar.

## Background

Since the emergence and spread of *Plasmodium falciparum *parasites resistant to inexpensive anti-malarial drugs, such as chloroquine (CQ) and sulphadoxine-pyrimethamine (SP), routine malaria case management has changed in endemic countries, such as Madagascar. Malaria diagnosis in these areas – particularly in zones not well covered by healthcare facilities – was entirely based on clinical examination, with CQ widely administered for any fever with no obvious alternative cause [1-3]. Since the introduction of more effective, more expensive anti-malarial drug combinations, such as artemisinin combination therapy (ACT), the WHO recommends the establishment of an accurate biological diagnosis before treatment and the withdrawal of presumptive anti-malarial treatment for all patients other than children under the age of five years in hyperendemic areas. This change in medical practice is now a public health priority in Africa, ensuring that effective anti-malarial drugs are administered only to the patients who need them and limiting the unnecessary use of inappropriate treatment, exposure to potential drug toxicity and the development of drug resistance.

The detection of *Plasmodium *species by the examination of blood films under a microscope remains the gold standard method for malaria diagnosis. However, this method, despite its simplicity and low cost, is not available everywhere. In peripheral health centres, in particular, there may be no appropriate microscope in working order or a lack of consumables, such as slides or Giemsa stain, or the staff may not have received appropriate training [4]. The development of alternative diagnostic tests for malaria, such as rapid diagnostic tests (RDTs), over the last ten years has made it possible to extend biological diagnosis to remote areas with few resources. These lateral-flow immunochromatographic tests detect specific antigens produced by malaria parasites and are rapid and simple to carry out, requiring no electricity or specific equipment [5-7]. A large number of branded RDT products are now available commercially (86 RDT products from 28 manufacturers), but all are based on the same principle and only three groups of antigen are detected. Most products detect *P. falciparum*-specific proteins, either *P. falciparum *histidine-rich protein 2 (PfHRP2) or *P. falciparum *lactate dehydrogenase (Pf-pLDH). Some detect both *falciparum*-specific and pan-specific antigens (aldolase or pan-malaria pLDH), distinguishing non-*falciparum *infections from *P. falciparum *or mixed infections.

Increase in the funding of malaria control has resulted in the National Malaria Control Programmes (NMCPs) of many endemic countries considering the possible use of RDTs in health facilities. The choice of an appropriate RDT is based on a number of factors, including sensitivity and specificity, stability, ease of use, cost, the prevalence of the various species infecting humans (choice between RDT detecting only *P. falciparum *or both *P. falciparum *and non-*falciparum *infections) and the format of the RDT. However, many factors, relating to either the parasite or the user, may affect the performance of RDTs for malaria [8,9]. Diversity in the genes encoding the proteins detected by the RDT may be a particularly important parasite factor. This has been demonstrated for PfHRP2, for which extensive diversity has been shown to affect the detection limits of RDTs based on PfHRP2 detection [10,11]. However, the degree of antigen variation has recently been shown to be limited for pLDH and parasite aldolase [12,13], suggesting that antigen polymorphism is unlikely to be responsible for sensitivity variation.

This study, designed to guide the NMCP in Madagascar in its choice of RDT for malaria, had the following objectives: (i) to assess the diversity of the *pfhrp2 *(and *pfhrp3*), *pldh *and *aldolase *genes in *P. falciparum *and *P. vivax *isolates from the various epidemiological strata for malaria in Madagascar and (ii) to determine the potential effect of this diversity on the performance of RDTs for malaria.

## Methods

### Study sites and parasite isolates

**Figure 1:**
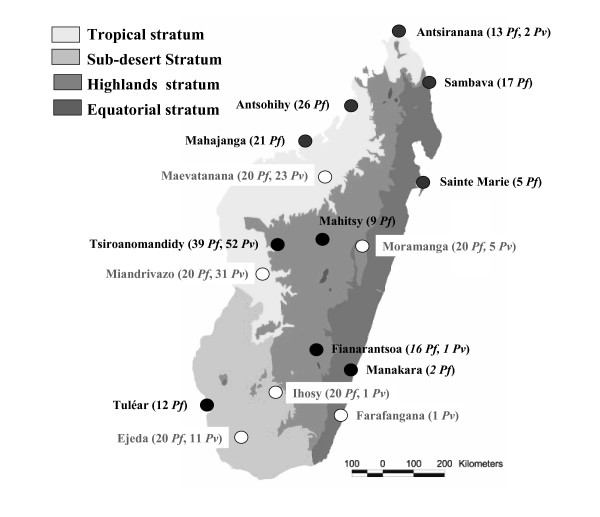
Geographic distribution of the 387 samples (260 *P. falciparum *and 127 *P. vivax*) collected in 2006 at 16 sites throughout Madagascar.

Two hundred sixty *P. falciparum *and one hundred twenty seven *Plasmodium vivax *isolates from 16 sites throughout Madagascar were collected in 2006 and analysed. These samples included 160 *P. falciparum *and 55 *P. vivax *isolates obtained from febrile inpatients at ten district hospitals and identified by microscopy. A further 172 samples (100 *P. falciparum *and 72 *P. vivax*) were collected from febrile outpatients attending six primary health centres. These patients tested positive for malaria with RDTs based on the detection of *Plasmodium*-specific lactate dehydrogenase (pLDH) (OptiMAL-IT™, DiaMed AG^©^, Cressier sur Morat, Switzerland). The diagnosis was confirmed by microscopy. These additional samples were not used for assessment of the genetic diversity of *Plasmodium *lactate dehydrogenase. All blood samples were stored dry spots on filter paper. The geographic distribution of the samples is shown in Figure 1.

### DNA extraction

DNA was extracted from blood spots, using Instagene^® ^Matrix resin (BioRad^©^, Marnes la Coquette, France), according to the manufacturer's instructions. The identification of parasite species was confirmed by real-time PCR, using species-specific primers, as previously described by de Monbrison [14], and a protocol adapted for use with the RotorGene^® ^3000 thermocycler (Corbett Life Science^®^, Sydney, Australia).

### Amplification by polymerase chain reaction

For *P. falciparum*, PCR amplification was carried out in a final reaction volume of 55 μl containing DNA template, primers (0.2 μM for each primer), dNTPs (Eurobio^© ^360 μM for each dNTP), 2.5 mM MgCl_2 _and 2.5 U FIREPol (Solis Biodyne^©^). The primers were designed to amplify exon 2 of the *Pfhrp2 *gene (PfHRP2-R and PfHRP2-F) and the *Pfhrp3 *gene (HRP3-R1 and HRP3-F2), exon 2 of the *Pf-aldolase *gene (Pf aldolase R1 and Pf aldolase F1) and the *Pf-lactate dehydrogenase *gene (PfLDH-R1 and PfLDH-F1). The primer sequences are shown in Table 1. PCR amplification conditions were identical for all four genes: denaturation at 94°C for 5 min, followed by 45 cycles of 94°C for 30 s, 57°C for 40 s and 72°C for 90 s, with a final extension phase at 72°C for 10 minutes.

**Table 1:** PCR primers, amplicon sizes and accession number of reference sequences used in the study.

**PCR primers named**	**PCR primers sequence 5' to 3'**	**Target gene**	**Amplicons size (bp)**	**GenBank accession number of reference sequences**	
				
				**Nucleotide**	**Protein**
PfHRP2-R	TTAATGGCGTAGGCAATGTG	*P. falciparum *histidine-rich protein II gene, exon 2	905	M13986	AAA51639
PfHRP2-F	TGTGTAGCAAAAATGCAAAAGG				
HRP3-R1	TGGTGTAAGTGATGCGTAGT	*P. falciparum *histidine-rich protein III gene	546	AF202093	AAF14632
HRP3-F2	AAATAAGAGATTATTACACGAAAG				
Pf aldolase R1	TTTCCTTGCCATGTGTTCAA	*P. falciparum *aldolase gene, exon 2	924	J03084	AAA29716
Pf aldolase F1	AGCAGATGTTGCCGAAGAAT				
PfLDH-R1	TTTCAGCTATGGCTTCATCAAA	*P. falciparum *lactate dehydrogenase gene	922	DQ825436	ABH03417
PfLDH-F1	GCACCAAAAGCAAAAATCGT				
Pvald PF	CCACTGGATCCGAATATAAA	*P. vivax *fructose 1,6-bisphosphate aldolase gene	1091	AF247063	AAK43741
Pvald PR	CTTTTCGTAAAGGGATGCT				
Pvald NF	CCTCAAACTACCCGCAGAAG		994		
Pvald NR	GGAGTTCGCTTCTGCTCTCT				
Pvldh PF	GTAGAGGCAGGTGAACACTC	*P. vivax *L-lactate dehydrogenase gene	1063	DQ060151	AAY59419
Pvldh PR	TCCTTTTAGTCTCCGCAAC				
Pvldh NF	AACCCAAAATTGTGCTCGTC		900		
Pvldh NR	CGTCGAACTTGGTCTTCTCC				

*Plasmodium vivax *DNA was amplified by nested PCR. The outer PCR was carried out in a final volume of 20 μl containing DNA template, 0.25 μM of each primer, 2.5 mM MgCl_2_, 200 μM of each dNTP (Eurobio^©^) and 1 U of FIREPol (Solis Biodyne^©^). The inner PCR was performed in a final reaction mixture of 55 μl containing PCR amplicons, 0.27 μM of each primer, 2.5 mM MgCl_2_, 360 μM of each dNTP (Eurobio^©^) and 2.5 U of FIREPol (Solis Biodyne^©^). The primer sequences used to amplify the *P. vivax fructose 1,6-bisphosphate aldolase *gene (outer primers, Pvald PF and Pvald PR; inner primers, Pvald NF and Pvald NR) and *P. vivax L-lactate dehydrogenase *gene (outer primers, Pvldh PF and Pvldh PR; inner primers, Pvldh NF and Pvldh NR) are shown in Table 1. After denaturation at 94°C for 4 minutes, the DNA was subjected to 40 cycles of 94°C for 20 s, 57°C for 20 s and 72°C for 80 s, with a final extension phase at 72°C for 10 minutes, for the outer PCR. Similar conditions were used for the inner PCR, but with different hybridization (58°C for 20 s) and elongation (72°C for 70 s) temperatures. Positive and negative controls were included in all assays (from the Malaria Research Reference Reagent Resource Center, MR4/ATCC, Manassas, Virginia: HRP II, MRA-67; HRP III, MRA-68, genomic DNA from *P. falciparum *3D7, MRA-102G and genomic DNA from *P. vivax *ONG, MRA-341G).

### Direct sequencing of PCR products

PCR products were visualised by agarose gel electrophoresis and purified by filtration through polyacrylamide P-100 Gel (Bio-Gel P-100, BioRad^®^, Marnes-la-Coquette, France) in 96-well plates (Millipore^®^, St. Quentin en Yvelines, France). Sequencing reactions were performed with BigDye Terminator chemistry (BigDye v 3.1 Terminator, Applied BioSystem^®^, Courtaboeuf, France) and a GeneAmp-9700 sequencer (Applied BioSystem^®^, Courtaboeuf, France). Sequencing was carried out with 10 μl of 0.3 mM EDTA par well, using an ABI 3700 automatic DNA sequencer (Applied BioSystems^®^, Courtaboeuf, France).

### Data analysis

Electrophoregrams were visualised and analysed with CEQTM 2000 Genetic Analysis System software (Beckman Coulter TM). Sequences of insufficient quality were either resequenced or rejected. Amino-acid sequences of the corresponding proteins were deduced from the nucleotide sequences obtained. Nucleotide and amino-acid sequences were compared with the reference sequences in Table 1, using Bio Edit Sequence Alignment Editor Software [15]. For *P. falciparum *histidine-rich proteins 2 and 3, the amino-acid repeat was identified by numerical code (1–18), as described by Baker *et al *[10].

### Statistical analysis

Based on the results of the analysis, isolates from the various sites were grouped into three distinct geographical regions: the North (Antsiranana, Sambava, Antosohihy, Mahajanga, Maevatanana and Sainte Marie), the Centre (Tsiroanomandidy, Miandrivazo, Mahitsy and Moramanga) and the South (Fianarantsoa, Manakara, Ihosy, Toliara and Ejeda).

The predictive model developed by Baker *et al. *was used to assess whether an isolate would be detected, if present at a density of ≤ 250 parasites/μl, by a RDT detecting PfHRP2 [10]. PfHRP2 sequences were classified into four groups as a function of the number of type 2 × type 7 repeats: group A (very sensitive) if the PfHRP2 sequence contained a more than 100 type 2 × type 7 repeats, group B (sensitive) if the number of type 2 × type 7 repeats was between 50 to 100, group C (non sensitive) if there were < 43 repeats and "borderline" group if the number of repeats was between 44 and 49.

Differences in the presence/absence of amino-acids repeats in isolates from different geographical areas were assessed by carrying out χ^2 ^tests, and differences in the mean number of amino-acid repeats between the three regions were assessed for each type of repeat, using the Kruskall-Wallis test (H test). *P *values < 0.05 were interpreted as indicating statistically significant differences.

## Results

### Polymorphisms in PfHRP2

For the 260 samples collected, 229 PCR *pfhrp2 *fragments were successfully amplified. These fragments ranged in size from 435 to 927 bp, giving proteins of 145 to 309 amino acids. Thirteen of the 14 different amino-acid repeats previously identified were detected [10]. Two hundred twenty one unique PfHRP2 sequences were identified, 213 of which were found in only one isolate and eight of which were found in two isolates. All PfHRP2 sequences had a similar structure: the type 1 repeat at the start (100% of the sequences), the central motif with the type 7, 8, 2, and 7 repeats combination (66.3% of the sequences) and the type 12 repeat at the end (100% of the sequences). Some repeats were observed in all PfHRP2 sequences (types 1, 2, 6, 7 and 12), whereas others were not observed (repeat 9) or were found in only a few sequences (repeat 8: 97.6%; repeat 3: 92.7%; repeat 10: 89.1%; repeat 5: 83.1%; repeat 4: 24.1%; repeat 13: 9.6%; repeat 14: 8.4% and repeat 11: 0.4%). No differences in the presence/absence of each repeat were found between the three geographic regions (*P *> 0.05). Further details are provided in Table 2.

**Table 2:** Distribution of amino-acid repeats in PfHRP2 and PfHRP3 from parasites collected throughout Madagascar in 2006.

**No. of amino acid repeats in PfHRP2 and PfHRP3**		Region and City															
			
		**North**						**Centre**				**South**					**Total**
					
		ATS	SBV	ATH	MJG	MAE	STM	TDD	MIA	MHT	MOR	FNR	MNK	IHO	TLR	EJE	
**1**	**AHHAHHVAD**	**1–5**	**1–5**	**1–6**	**1–5**	**1–5**	**2**	**1–5**	**1–4**	**1–3**	**1–4**	**2–4**	**3**	**1–4**	**1–4**	**1–4**	**1–6**
*1*	*AHHAHHVAD*	*1*	*1–2*	*1–3*	*1–3*	*1*	*1*	*1–3*	*1–3*	*1–2*	*1*	*1–2*	*1*	*1–4*	*1*	*1*	*1–4*
**2**	**AHHAHHAAD**	**8–16**	**11–13**	**10–14**	**4–15**	**8–14**	**14**	**10–14**	**11–13**	**11–12**	**10–13**	**8–15**	**12**	**8–14**	**7–14**	**7–15**	**4–16**
**3**	**AHHAHHAAY**	**0–2**	**1–2**	**1–2**	**0–2**	**0–2**	**0**	**1–2**	**1–2**	**1–2**	**1–2**	**1–2**	**0**	**1–2**	**1–3**	**0–3**	**0–3**
**4**	**AHH**	**0–1**	**0–1**	**0–1**	**0–3**	**0–2**	**0**	**0–1**	**0–1**	**0–1**	**0–1**	**0**	**1**	**0**	**0–1**	**0–1**	**0–3**
*4*	*AHH*	*1*	*1*	*1–2*	*1*	*1*	*1*	*1*	*1*	*1*	*1*	*1*	*1*	*1*	*1*	*1*	*1–2*
**5**	**AHHAHHASD**	**0–1**	**1**	**0–3**	**0–1**	**0–1**	**1**	**0–2**	**0–2**	**1**	**0–1**	**0–3**	**0**	**0–2**	**1–2**	**0–2**	**0–3**
**6**	**AHHATD**	**3–5**	**3–5**	**2–5**	**2–7**	**2–6**	**2**	**2–8**	**2–6**	**3–5**	**3–8**	**1–3**	**3**	**1–3**	**2–4**	**2–5**	**1–8**
**7**	**AHHAAD**	**2–10**	**4–7**	**4–11**	**3–8**	**4–8**	**6**	**3–9**	**3–8**	**5–8**	**3–10**	**4–13**	**6**	**5–10**	**4–9**	**3–8**	**2–13**
*7*	*AHHAAD*	*1*	*1*	*1*	*1*	*1*	*1*	*1*	*1*	*1*	*1*	*1*	*1*	*1*	*0–1*	*1*	*0–1*
**8**	**AHHAAY**	**1–2**	**1–2**	**1–2**	**1–2**	**1–2**	**1**	**0–2**	**0–2**	**1–2**	**0–2**	**0–2**	**3**	**0–2**	**1–2**	**1–2**	**0–2**
**9**	**AAY**	**0**	**0**	**0**	**0**	**0**	**0**	**0**	**0**	**0**	**0**	**0**	**0**	**0**	**0**	**0**	**0**
**10**	**AHHAAAHHATD**	**0–2**	**0–2**	**1–3**	**1–3**	**1–2**	**2**	**0–2**	**0–2**	**2–3**	**1–2**	**0–2**	**1**	**1–2**	**0–2**	**0–2**	**0–3**
**11**	**AHN**	**0**	**0**	**0**	**0–1**	**0**	**0**	**0**	**0**	**0**	**0**	**0**	**0**	**0**	**0**	**0**	**0–1**
**12**	**AHHAAAHHEAATH**	**1**	**1**	**1**	**1**	**1**	**1**	**1**	**1**	**1**	**1**	**1**	**1**	**1**	**1**	**1**	**1**
**13**	**AHHASD**	**0–1**	**0–2**	**0–1**	**0**	**0–1**	**0**	**0–1**	**0–1**	**0**	**0–1**	**0**	**0**	**0–1**	**0**	**0–1**	**0–2**
**14**	**AHHAHHATD**	**0–2**	**0**	**0–1**	**0–1**	**0–1**	**0**	**0–1**	**0–1**	**0–1**	**0–1**	**0**	**0**	**0–1**	**0–1**	**0–1**	**0–2**
*15*	*AHHAAY*	*1*	*1*	*1*	*1*	*1*	*1*	*1*	*0–1*	*1*	*1*	*1*	*1*	*1*	*1*	*1*	*0–1*
*16*	*AAY*	*8–14*	*8–13*	*7–13*	*9–13*	*8–18*	*10–14*	*10–14*	*10–16*	*8–14*	*8–15*	*8–14*	*15*	*10–14*	*8–15*	*7–14*	*7–18*
*17*	*AHHAAAHHATD*	*1–8*	*5–9*	*5–8*	*4–7*	*5–8*	*4–8*	*5–7*	*3–7*	*5–10*	*0–7*	*4–10*	*6*	*4–8*	*4–6*	*3–8*	*0–10*
*18*	*AHN*	*1–3*	*1–4*	*1–3*	*1–4*	*1–3*	*2–3*	*1–3*	*1–3*	*2*	*0–3*	*2*	*2*	*1–2*	*1–3*	*2–3*	*0–4*

Significant differences between the number of repeats as a function of geographic region were seen for repeats 5 and 6: isolates from the South had significantly larger numbers of type 5 repeats than isolates from the North and Centre and fewer type 6 repeats than isolates from the North and Centre (Table 3).

**Table 3:** Comparison of the mean number of each type of repeat in PfHRP2 and PfHRP3 from parasites from different geographic areas.

	**PfHRP2 sequences**									**PfHRP3 sequences**								
		
**Type of repeats**	**Total (n = 229)**		**North (n = 90)**		**Centre (n = 81)**		**South (n = 58)**		***P***	**Total (n = 237)**		**North (n = 90)**		**Centre (n = 84)**		**South (n = 63)**		***P***
									
	**mean**	**SD**	**mean**	**SD**	**mean**	**SD**	**mean**	**SD**		**mean**	**SD**	**mean**	**SD**	**mean**	**SD**	**mean**	**SD**	
**1**	**2.6**	**1.2**	2.7	1.4	2.3	1.0	2.66	1.17	0.58	**1.1**	**0.4**	1.1	0.4	1.1	0.4	1.2	0.5	0.70
**2**	**12.0**	**1.8**	12.1	2.1	12.1	1.1	11.5	2.3	0.81									
**3**	**1.4**	**0.6**	1.2	0.7	1.5	0.5	1.5	0.6	0.15									
**4**	**0.3**	**0.5**	0.3	0.6	0.3	0.5	0.1	0.4	0.47	**1.0**	**0.1**	1.0	0.1	1.0	0	1.0	0	0.36
**5**	**0.9**	**0.6**	0.8	0.5	0.9	0.4	1.3	0.7	0.03									
**6**	**3.4**	**1;2**	3.5	1.1	3.8	1.4	2.7	0.8	0.01									
**7**	**6.0**	**2.0**	6.0	1.9	6.1	1.7	6.2	2.3	0.73	**1.0**	**0.1**	1.0	0	1.0	0	0.98	0.14	0.31
**8**	**1.1**	**0.5**	1.3	0.4	1.1	0.4	1.3	0.6	0.23									
**10**	**1.5**	**0.8**	1.6	0.7	1.6	0.8	1.4	0.8	0.71									
**13**	**0.3**	**0.9**	0.2	0.4	0.04	0.2	0,0	0,0	0.08									
**14**	**0.1**	**0.3**	0.1	0.4	0.1	0.3	0.1	0.3	0.8									
**15**										**1.0**	**0.1**	1.0	0	1.0	0.1	1.0	0	0.43
**16**										**11.4**	**1.8**	11.2	1.5	11.5	1.9	11.4	1.8	0.73
**17**										**5.8**	**1.3**	5.7	1.3	5.7	1.3	6.0	1.3	0.68
**18**										**2.1**	**0.5**	2.1	0.6	2.1	0.5	2.0	0.4	0.61

Based on the model of Baker *et al*, the frequency of non sensitive isolates (Group C, not detected for parasitaemia ≤ 250 parasites/μl) were predicted to be 9%. No significant differences were found between the three regions in the frequency of the four groups of Baker's model (*P *> 0.05) (Table 4).

**Table 4:** Prediction of RDT detection sensitivity based on the regression model of Baker *et al *(1).

**Region of Madagascar**	**No. of type 2 × type 7 repeats goup (%)**				
	
	**Group A**	**Group B**	**"Borderline" Group**	**Group C**	**Total**
**North**	11 (12)	65 (72)	9 (10)	5 (6)	90
**Centre**	4 (5)	57 (70)	12 (15)	8 (10)	81
**South**	7 (12)	36 (62)	7 (12)	8 (14)	58
					
**Total**	22 (10)	158 (69)	28 (12)	21 (9)	229

### Polymorphisms in the PfHRP3 gene

For PfHRP3, 238 PCR fragments were successfully amplified. They ranged in size from 405 to 720 bp and from the corresponding protein sequences were 135 to 240 amino acids long. Seven of the eight previously described amino-acid repeats were identified [10]. One hundred five unique PfHRP3 sequences were identified: 60 were observed only once, 25 were observed twice, four sequences were shared by three or seven isolates, three sequences were found in four, five and twelve isolates and one sequence was present in six, eight and eleven isolates. All PfHRP3 sequences started with the type 1 repeat and ended with a combination of the type 17 and type 4 repeats.

All repeats found in Madagascar were observed in all PfHRP3 sequences (types 1, 4, 7, 15, 16, 17, 18). No differences in the presence/absence of each repeat were found between the three geographic regions (*P *> 0.05). Further details are provided in table 2. No significant difference in the number of repeats was observed between the different geographic regions (Table 3).

### Polymorphisms in the aldolase gene

Comparisons of 240 *P. falciparum *aldolase gene sequences obtained from 260 isolates collected throughout Madagascar showed a high level of conservation. Only four single nucleotide polymorphisms (SNP) were observed in six isolates (Table 5). The two parasites with the SNP at nucleotide 815 (amino-acid change at codon 104: H to R) originated from the North (Antsohihy and Maevatanana), whereas the two parasites with the SNP at nucleotide 1404 (amino-acid change at codon 300: H, basic polar amino acid to Q, neutral polar amino acid) originated from the North (Antsiranana) and South (Ejeda). The other two SNPs found in one isolate originated from the South: Ihosy for the SNP at nucleotide 1314 (non synonymous change) and Ejeda for the SNP at nucleotide 1417 (amino-acid change at codon 305: T, neutral polar amino acid to A, neutral non-polar amino acid).

**Table 5:** SNPs and amino-acids changes in aldolases and pLDHs in *P. falciparum *and *P. vivax *isolates collected in Madagascar in 2006, from different geographic areas.

**Genes**	**SNP (bp position and change)**	**Amino acid change (aa position and change)**	**Frequency No (%)**
***P. falciparum *aldolase**	815 (CAC to CGC)	104 (H to R)	2 (0.8)
	1314 (CCA to CCC)	synonymous change	1 (0.4)
	1404 (CAC to CAG)	300 (H to Q)	2 (0.8)
	1417 (ACC to GCC)	305 (T to A)	1 (0.4)

***P. vivax *aldolase**	510 (TCC to TCA)	synonymous change	1 (0.9)
	651 (TTA to TTG)	synonymous change	43 (39.1)

***P. falciparum LDH***	73 (CAG to AAG)	25 (Q to K)	1 (0.7)
	814 (GAT to AAT)	272 (D to N)	10 (7.3)

***P. vivax LDH***	168 (ATG to ATA)	56 (D to N)	1 (0.9)
	225 (AAG to AAA)	synonymous change	84 (75.7)
	405 (ATC to ATT)	synonymous change	2 (1.8)
	498 (GTC to GTT)	synonymous change	1 (0.9)
	544 (GTT to ATT)	182 (V to I)	1 (0.9)
	627 (GTG to GTC)	synonymous change	32 (28.8)
	687 (GCC to GCT)	synonymous change	23 (20.7)

For the 110 *P. vivax *aldolase gene sequences, only two synonymous changes were observed. The SNP at nucleotide 510 were found in one isolate from the Centre (Tsiroanomandidy) whereas the SNP at nucleotide 651 was observed in 43 isolates from the three regions (Table 5).

### Polymorphisms in the pLDH gene

Two SNPs were observed among the 137 DNA sequences obtained from the 160 *P. falciparum *isolates. The SNP at nucleotide 73 (amino-acid change at codon 25: Q, neutral polar amino acid to A, basic polar amino acid) was found in one isolate from Tsiroanomandidy. In addition, 10 isolates displayed a change of nucleotide at position 814 with respect to the reference sequence, resulting in an amino acid change (D, acidic polar amino acid to N, neutral polar amino-acid). These isolates came from the Centre (Mahitsy and Tsiroanomandidy) and the South (Fianarantsoa and Tulear) (Table 5).

Greater diversity in the aldolase gene was observed for *P. vivax*, with seven different nucleotide changes, including two non-synonymous amino-acid changes. Three synonymous amino-acid changes were frequent and found in all three geographic regions (nucleotide changes at nucleotides 225, 627 and 687). The other four nucleotide changes were rare. The change at nucleotide 405 was displayed by two isolates, one from Miandrivazo and one from Maevatanana, and the change at nucleotide 168 (amino acid change from V to I, two neutral non polar amino acids) was displayed by one isolate from Ejeda. The other two changes were observed in two isolates from Miandrivazo: at nucleotide 168 (amino-acid change from D, acidic polar amino acid to N, neutral polar amino acid) and at nucleotide 498 (Table 5).

## Discussion

With the abandon of the inexpensive anti-malarial drug treatments, such as chloroquine, and the use of ACT as a first-line treatment, parasitological confirmation of suspected cases of malaria has become essential. RDTs are now indispensable tools in malaria management, particularly in countries with endemic malaria in which health structures are under-equipped. The WHO has issued some recommendations, but the choice of RTD remains difficult for most users in endemic countries [16,17]. Indeed, in addition to traditional considerations, such as the *Plasmodium *species to be detected, the stability of storage and use conditions, ease of use and cost, other technical aspects must be taken into account. One of the most important, but least studied of these factors is genetic variability of the antigens detected by the antibody component of the RTD. Indeed, very few data are currently available, and the available data relate only to continental level [10-13]. There is, therefore, clearly a need for more data on pLDH and aldolase antigen variation and the geographic extent of HRP2 variation, especially at local level.

This study provides the first country-wide evaluation of genetic polymorphism for the *P. falciparum *and *P. vivax *antigens detected by RDTs for malaria, these two species being the principal species of *Plasmodium *species found in Madagascar [18-20].

Consistent with previous reports [10], the PfHRP2 antigen (and the PfHRP3 antigen) was found to be highly diverse in parasite isolates throughout Madagascar. However, No significant differences, other than the number of repeats for repeats 5 (AHHAHHASD) and 6 (AHHATD), were found in the PfHRP2 and PfHRP3 sequences in the three distinct geographic regions. The sequences obtained were compared with others from around the world [10]. Malagasy HRP2 sequences had certain characteristics in common with African isolates, particularly those from Cameroon (presence of repeats 11, 13 and 14), and with Asian/Pacific isolates, especially those from Thailand (presence of repeats 13 and 14), the Philippines (presence of repeat 11), Papua New Guinea (presence of repeat 14) and the Solomon Islands (presence of repeats 11 and 14), probably reflecting the diversity of populations currently living in Madagascar but originating from Africa, India, Asia, the Middle East and Europe [21].

Finally, Baker's regression model showed that only 91% of the *P. falciparum *parasites were likely to be detected by RDTs based on HRP2 detection at parasite densities ≤ 250 parasites/μl. We found that the predicted prevalence of parasites undetectable at densities ≤ 205 parasites/μl ("non-sensitive" parasites) was intermediate between that in Asia-Pacific (mean of 23%, range from 17% in South-East Asia to 33% in Papua New Guinea) and that in Africa or South America (0%). We also found that the prevalence of non-sensitive isolates in Madagascar was higher in the South (14%) than in the North (6%), the Centre having an intermediate prevalence (10%). However, this trend was not statistically significant (*P *< 0.17). One of the major issues linked to the high prevalence of non-sensitive strains in southern Madagascar – an epidemic-prone area – is the high proportion of individuals with low levels of immunity in this region and likely to present clinical symptoms even with a parasitaemia below 250 parasites/μl, a level not generally considered life-threatening. The use of RTDs based on PfHRP2 detection would probably give false negative results in 10% to 20% of cases, leaving patients untreated and therefore at risk of developing severe complications. Indeed, parasitaemia undetectable with RDTs might well occur among the non-immune tourists visiting Madagascar, who would then probably go on to develop clinical symptoms on their return to Europe.

Unlike PfHRP2 (and PfHRP3), the *P. falciparum *and *P. vivax *aldolase and pLDH genes appear to be highly conserved among the Malagasy isolates sequenced, confirming published data [12,13]. Indeed, for the aldolase gene, none of the *P. vivax *samples displayed an amino-acid change and only three previously undescribed amino-acid changes were identified for *P. falciparum *samples (at codons 104, 300 and 305). Similarly, we observed only four amino-acid changes in the *pLDH *gene, two in *P. falciparum *isolates (at codons 25 and 272) and two in *P. vivax *samples (at codons 56 and 182). Most *P. vivax *isolates displayed nucleotide differences with respect to the sequence of the reference strain used: 39.1% of Malagasy isolates displayed a change at nucleotide 651 with respect to the Belem strain aldolase gene sequence (accession no. DQ060151), and 75.7% displayed a change at nucleotide 225, 28.8% at nucleotide 627 and 20.7% at nucleotide 687, with respect to the WDK strain pLDH gene sequence (accession no. AF247063). There are two possible explanations for this difference: sequencing errors for the reference strains used in this study or a specific feature of the Malagasy isolates, these SNPs having never been described before.

## Conclusion

The findings of the present study confirm previous data concerning the greater polymorphism of PfHRP2 and PfHRP3 antigens than of *P. falciparum *and *P. vivax *aldolase and pLDH antigens. Based on analysis of the PfHRP2 sequences from Madagascar, we predict that 9% of the Malagasy isolates would not be detected at densities ≤ 250 parasites/μl, despite the recommendation to use RDTs based on PfHRP2 detection in the health facilities of the 111 sanitary districts. Thus, in countries using this type of RDT for PfHRP2 sequence detection, efforts should be made to determine more accurately the prevalence of non-sensitive parasites. The results of this study suggest that the National Malaria Control Programme in Madagascar should train its healthcare staff and the end-users of RDTs to provide information concerning the possibility of false-negative results for patients with clinical symptoms of malaria, particularly in South Madagascar. RDTs based on pLDH detection may also be a useful alternative to HRP2-based RDTS, despite their lower sensitivity [22] and stability [23].

### Nucleotide sequence accession numbers

The nucleotide sequences reported here have been deposited in the GenBank database under accession numbers EU589688 to EU589767 for *P. falciparum hrp-2 *sequences, EU589768 to EU589942 for *P. falciparum hrp-3 *sequences, EU589943 to EU589946 for *P. falciparum *aldolase gene sequences, EU589947 to EU589948 for *P. falciparum pLDH *sequences, EU589951 to EU589957 for *P. vivax pLDH *sequences and EU589949 to EU589950 for *P. vivax *aldolase gene *sequences*.

## Competing interests

The authors declare that they have no competing interests.

## Authors' contributions

CBa and NM performed laboratory work and wrote the manuscript. MT and CBo carried out sequencing and gave constructive advice. DM was involved in all stages of this study.
